# Voltage-dependent Ca^2+^ channels, not ryanodine receptors, activate Ca^2+^-dependent BK potassium channels in human retinal pigment epithelial cells

**Published:** 2008-12-15

**Authors:** Sönke Wimmers, Claire Halsband, Sebastian Seyler, Vladimir Milenkovic, Olaf Strauß

**Affiliations:** 1 Experimentelle Ophthalmologie, Klinik und Poliklinik für Augenheilkunde, Universitätsklinikum Hamburg-Eppendorf, Hamburg, Germany; 2 Experimentelle Ophthalmologie, Klinik und Poliklinik für Augenheilkunde, Klinikum der Universität Regensburg, Regensburg, Germany; 3 Institut für Neurophysiologie, Medizinische Hochschule Hannover, Hannover, Germany

## Abstract

**Purpose:**

In different tissues the activation of large conductance Ca^2+^-activated (BK) potassium channels has been shown to be coupled to voltage-gated Ca^2+^ channels as well as ryanodine receptors. As activation of BK channels leads to hyperpolarization of the cell, these channels provide a negative feedback mechanism for Ca^2+^-induced functions. Many cellular functions of the retinal pigment epithelium (RPE) are coupled to changes in [Ca^2+^]_i_. The aim of this study was to identify which Ca^2+^-entry pathway leads to the activation of BK channels in the RPE.

**Methods:**

We used freshly isolated human RPE cells and the ARPE-19 cell line for the detection of transcripts of BK channel α subunits. Patch-Clamp measurements were used to characterize BK channels in ARPE-19 cells electrophysiologically. To monitor changes in [Ca^2+^]_i_ ARPE-19 cells were loaded with Fura-2.

**Results:**

Freshly isolated human RPE cells and ARPE-19 cells were shown to express BK channels. In ARPE-19 cells these channels were shown to be functionally active. Application of iberiotoxin led to a block of outward currents by 28.15%. At +50 mV ARPE-19 cells had a BK channel-mediated current density of 2.42 pA/pF. Activation of ryanodine receptors by caffeine led to a significant increase in [Ca^2+^]_i_ by 34.16%. Nevertheless, caffeine-induced Ca^2+^ signals were not sufficient to activate BK channels. Instead, the activation of L-type Ca^2+^ channels by BayK 8644 caused a dramatic increase in BK channel activity and a shift of the reversal potential of the ARPE-19 cells by −22.6 mV.

**Conclusions:**

We have shown here for the first time that human RPE cells express BK channels. These channels are activated in RPE cells by increases in [Ca^2+^]_i_ that are mediated by the opening of voltage gated L-type Ca^2+^ channels. As Ca^2+^ entering the RPE cells through these Ca^2+^ channels are known to be important for growth factor secretion and light-induced transepithelial transport, we speculate that BK channels coupled directly to these Ca^2+^ channels may provide a good tool for negative feedback control of the L-type Ca^2+^ channels.

## Introduction

Large conductance Ca^2+^-activated (BK) channels take a special position within the family of K^+^ channels in that their open probability is increased by either membrane depolarizations or increases in the intracellular free Ca^2+^ concentration ([Ca^2+^]_i_). Accordingly, BK channels may serve as negative feedback regulators for events that depolarize the cell or lead to raises in [Ca^2+^]_i_. In neurons, they have been shown to control secretion of neurotransmitters in cooperation with voltage-gated Ca^2+^ channels [1] or to control action potential frequency [2]; in vascular smooth muscle cells, they regulate the contractile tone [3]; and in chromaffin cells, their activation regulates excitability by generation of afterhyperpolarizations [4].

In the retinal pigment epithelium (RPE), changes in [Ca^2+^]_i_ regulate a variety of cell functions: dark adaptation of photoreceptor activity, transepithelial transport, phagocytosis, growth factor secretion, and differentiation [5]. [Ca^2+^]_i_, in turn, is controlled by different transporters and channels. Intracellular Ca^2+^ stores are depleted by activation of inositol 1,4,5-trisphosphate and ryanodine receptors [6]. In the cell membrane, functionally active voltage-gated Ca^2+^ channels [7–11] and classical transient receptor potential channels (TRPC; [12,13]) have been detected. In addition, ATP-stimulated rises in intracellular free Ca^2+^, possibly resulting from the activation of ionotropic purinergic receptors (P2X), have been shown [14]. While TRPC have been demonstrated to be constitutively active [13], depletion of intracellular Ca^2+^ stores and activation of voltage-gated Ca^2+^ channels has been linked to various stimuli [15–19]. As all these Ca^2+^ pathways lead to an increase in [Ca^2+^]_i_, they all potentially activate BK channels. Until now, BK channels in the RPE have been shown to be activated by oxidizing agents [20], by hypotonicity and consequently cell swelling [21], by mechanical stress [22], and by increases in [Ca^2+^]_i_ [23]. But nothing is known about the Ca^2+^ source responsible for increases in [Ca^2+^]_i_ that leads to the activation of BK channels in the RPE. The aim of this study was to identify which Ca^2+^ entry pathway leads to the activation of BK channels in the RPE.

## Methods

### Cell culture

The human retinal pigment epithelial cell line ARPE-19 (ATCC, Manassas, VA) was cultured in Dulbecco’s modified eagle medium:F-12 nutrient mixture (Invitrogen, Karlsruhe, Germany), which contained 10% fetal bovine serum (Invitrogen), 0.05% insulin-transferrin-sodium (Roche, Mannheim, Germany), 1% nonessential amino acids (Invitrogen), 100 U/ml penicillin and 0.1 mg/ml streptomycin (both Invitrogen). Cells were cultured at 37 °C in a humidified ambient atmosphere containing 5% CO_2_ and were passaged twice a week. For RNA isolation and Ca^2+^ measurements, confluent cultures of ARPE-19 cells were used up to passage number 75. For electrophysiological measurements, cells were passaged the day before the experiments were conducted to get isolated single cells.

### RNA isolation and RT PCR

Human RPE was obtained from organ donors within 24 h of death. The human donor eyes were obtained after corneal transplantation from the cornea tissue bank of the Eye Hospital at the University Hospital Hamburg-Eppendorf. For the use of human material, tenets of the Declaration of Helsinki were followed, informed consent was obtained from the relatives of the donors, and Institutional Human Experimentation Committee approval was granted for the studies. After the cornea was removed for transplantation, whole eyes without cornea were delivered ice-cold and processed for RPE preparation. The anterior parts of the eyes, including the vitreous and the retina, were removed. The posterior portion was rinsed with ice-cold D-PBS without Ca^2+^ and Mg^2+^ (Invitrogen) to wash away residuals of the neuronal retina. A fine pair of forceps was used to gently brush away the RPE. The RPE cells were collected and lysed in the lysis buffer of the RNeasy Mini Kit (Qiagen, Hilden, Germany). Total RNA from ARPE-19 cells was prepared from confluent cultures grown in a 25 cm^2^ culture flask. RNA was isolated using the RNeasy Mini kit (Qiagen) according to the manufacturer’s instructions. Next, 1 µg RNA was reverse transcribed at 37 °C for 1 h in the following reaction mixture: 1 µg oligo dT primer (Invitrogen), 1 mM of each dNTP, 20 U RNAguard (Amersham Biosciences Europe, Heidelberg, Germany), and 20 U Moloney Murine Leukemia Virus (M-MLV) reverse transcriptase (Invitrogen). For control PCR reactions human total brain RNA (Stratagene Europe, Amsterdam, Netherlands) was reverse transcribed under the same conditions. PCR experiments were performed with 1 µl cDNA in 50 µl PCR reaction mixtures with Taq DNA polymerase (Stratagene). The following oligonucleotides specific for the BK channel gene *kcnma1* (NM_002247) were used: forward GGA ATG GGA GAC GCT TCA TA, reverse CCT GCA GCG AAG TAT CAT CAT CA. For amplification, we used 40 cycles as follows 30 s at 95 °C, 30 s at 60 °C, and 60 s at 72 °C. The identity of the amplification product was confirmed with agarose (1.5%) gel electrophoresis with O'RangeRuler™ 100 bp DNA Ladder (Fermentas, St. Leon-Roth, Germany) and by sequencing using the Big Dye Terminator^TM^ kit (Perkin Elmer Applied Biosystems, Weiterstadt, Germany). RT PCR experiments were repeated 3 times.

### Electrophysiology

ARPE-19 cells were placed in a bath chamber on the stage of an Axiovert 35 inverted microscope (Zeiss, Goettingen, Germany). The bath solution was composed of 130 mM NaCl, 5 mM KCl, 2 mM MgCl_2_, 2 mM CaCl_2_, 10 mM HEPES, 5 mM glucose, adjusted to pH 7.3 with NaOH. Patch-clamp electrodes with a resistance of 3–5 MΩ were pulled from borosilicate glass using a DMZ Universal Puller (Zeitz-Instruments, Martinsried, Germany). The electrodes were filled with a solution containing 140 mM KCl, 2 mM MgCl_2_, 1 mM CaCl_2_, 2.5 mM EGTA, 10 mM HEPES, adjusted to pH 7.3 with KOH. Whole-cell currents were measured using an EPC-9 (Heka, Lambrecht, Germany) patch-clamp amplifier in conjunction with TIDA software (Heka) for data acquisition and analysis. Fast and slow capacity transients were compensated. Series resistance errors were compensated to at least 75%. All experiments were performed at room temperature (22–25 °C).

### Measurement of intracellular free Ca^2+^ concentrations

The ARPE-19 cells grown on coverslips to confluency were washed with Krebs Henseleit solution, which contained 118 mM NaCl, 5 mM KCl, 1.2 mM MgCl_2_, 1.8 mM CaCl_2_, 1.2 mM NaSO_4_, 2 mM NaH_2_PO_4_, 9 mM glucose, and 20 mM HEPES, adjusted to pH 7.4 with NaOH. Cells were then loaded with Fura-2 AM ester (Fluka, Buchs, Switzerland) for 40 min in the dark at room temperature in Ringer solution containing 10 µM Fura-2 AM Next, cells were washed and then incubated for at least 30 min with Krebs Henseleit solution. The coverslips were placed into a bath chamber perfused constantly with Krebs Henseleit solution and mounted onto a Zeiss inverted microscope (Axiovert 35) equipped with a 40X Fluar objective. Substances were applied extracellularly by the perfusion system. In control experiments [Ca^2+^]_i_ was measured during changes between running and arrested perfusion. The absence of changes in [Ca^2+^]_i_ during this maneuver indicated no influence of mechanosensitive channels when using the perfusion for drug application. Furthermore, in experiments which aim to investigate the role of cytosolic Ca^2+^ stores 50 mM caffeine was applied. Analysis of the cell size during caffeine application revealed no hyperosmotic shrinkage of cells: 100% before caffeine application; 99.87%±0.28% (mean±SEM; n=16) at the end of caffeine application. We performed ratiometric measurements Fura-2 fluorescence at 5 s intervals using a high-speed polychromator system (Visitron Systems, Puchheim, Germany) altering the wavelength of excitation light between 340 and 380 nm. Emitted light was filtered with a 510 nm filter and detected by a cooled charged-coupled device camera (CoolSNAP, Visitron Systems). Data were collected with MetaFlour software (Molecular Devices, Downington, PA) and analyzed with MetaAnalysis software (Molecular Devices). To calculate intracellular free Ca^2+^ ([Ca^2+^]_i_), we superfused cells with the Krebs Henseleit solution without Ca^2+^ supplemented with 1 mM EGTA and 1 µM ionomycin. The cells were then superfused with Krebs Henseleit solution supplemented with 1 µM ionomycin and saturating Ca^2+^ concentration. [Ca^2+^]_i_ was calculated according to Grynkiewicz et al. [24] [Ca^2+^]=K_d_ *(R-R_min_)/(R_max_-R)*S_f_/S_b_. Where R is the fluorescence intensity ratio (F(340)/F(380)), R_min_ the value where R is minimal (with EGTA), R_max_ the value when R is maximal (with saturating Ca^2+^ and ionomycin). K_d_ is the dissociation constant of Fura-2 and S_f_ and S_b_ the maximal respectively minimal fluorescence after excitation with 380 nm.

### Data analysis

Half-maximal activation was obtained from a fit with a Boltzmann equation:

I=a/{1+exp[-(V-V_1/2_)/k]},

where V_1/2_ is the potential of half-maximal activation and k is the slope factor. Activation time constants were calculated by fitting current traces by a mono-exponential function:

I=a+b* exp(τ/c),

where τ is the time constant. Results were presented as mean ±SEM. Statistical significance was tested using one-way ANOVA. n=number of independent experiments, * stands for statistical significance with p<0.05.

## Results

**Figure 1:**
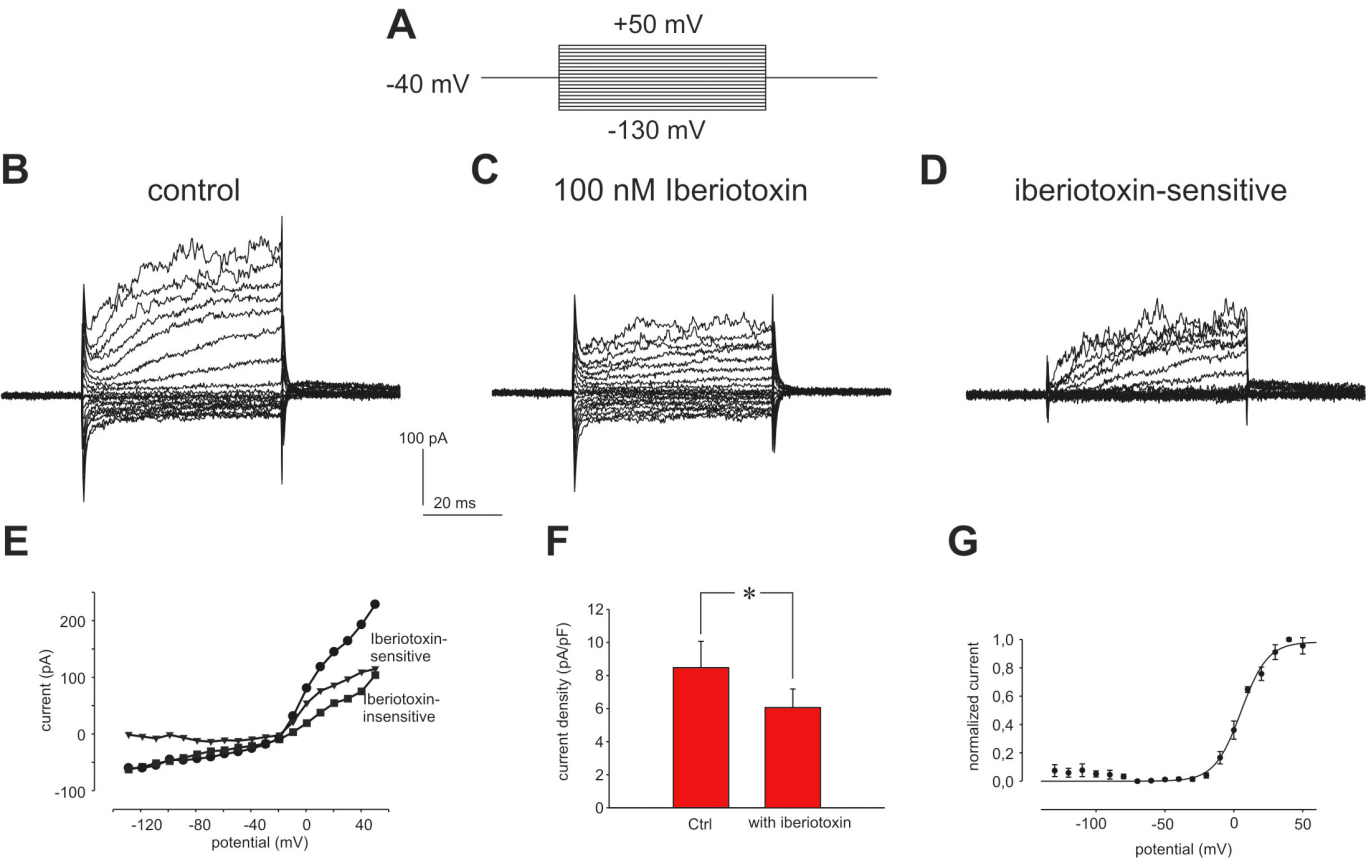
Electrophysiological measurements of BK channels in ARPE-19 cells. **A:** Shown is the pulse protocol used to measure Ca^2+^-activated potassium currents. From a holding potential of −40 mV the cells were stimulated by 20 potential steps of 50 ms duration from −130 mV with 10 mV increment. **B:** Shown are control currents evoked in Ringer solution by the pulse protocol shown in **A**. **C:** Shown is the same cell as in **A** in the presence of the specific BK channel antagonist iberiotoxin (100 nM). **D:** Shown is the iberiotoxin-sensitive current of the same cell (**A-B**). **E:** The current-voltage relationship of the steady-state current displayed here, illustrates that only outward current was blocked by iberiotoxin. Shown are the data from the cell measured in **A-C**. Abbreviations: Control without iberiotoxin represents ●; iberiotoxin-insensitive represents ■; iberiotoxin-sensitive represents ▼. **F:** The comparison of current densities at +50 mV measured before and after application of 100 nM iberiotoxin illustrates the reduction of outward currents by the addition of the blocker (n=6). **G:** Shown here is the current-voltage relationship of normalized mean iberiotoxin-sensitive currents fitted with a Boltzmann equation (V1/2=5.23 mV, k=9.58 mV, n=4).

A typical recording of outwardly rectifying whole cell currents from ARPE-19 cells is shown in Figure 1B. The cells had a mean current density at +50 mV of 8.47±1.6 pA/pF (n=6; Figure 1F). Application of the specific BK channel blocker iberiotoxin reduced the outward current significantly by 28.15±7.14% without affecting inward currents (Figure 1B-E). The iberiotoxin-sensitive current activated at potentials positive to −10 mV and reached half-maximal activation at 4.73±1.68 mV (n=4; Figure 1G). Currents evoked by voltage-steps to +50 mV activated with a time constant of 10.36±1.34 ms.

**Figure 2:**
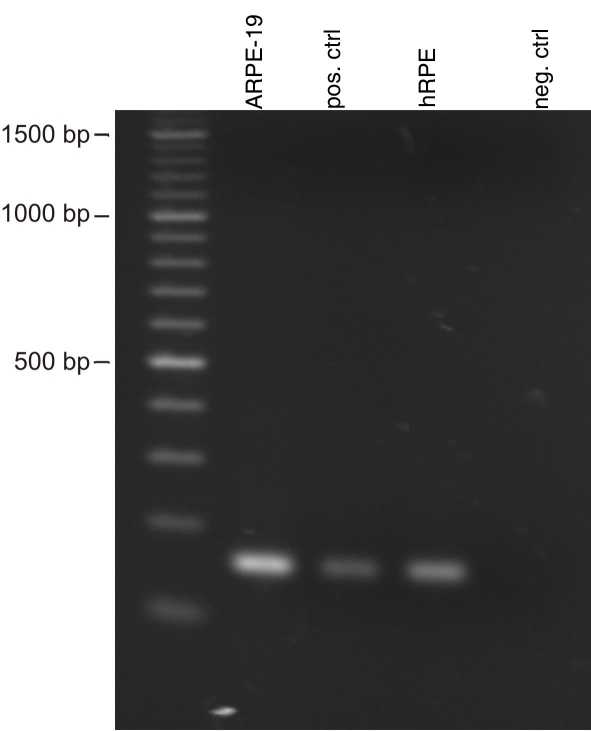
Expression of the BK channel α subunit by the RPE. Displayed is an RT–PCR experiment showing the expression of the α subunit of BK channels in the ARPE-19 cell line and in freshly isolated human RPE tissue. As positive control we used fetal human brain cDNA and as negative control water instead of cDNA. The sizes of the major marker bands are indicated.

Although the presence of BK channels in RPE cells has been demonstrated in cultivated rabbit RPE cells and in another human RPE cell line (RPE-50), their expression has not yet been demonstrated at the mRNA or protein level. In Figure 2 the results of RT PCR experiments using mRNA from the ARPE-19 cell line and from freshly isolated human RPE cells are shown. These results demonstrate that BK channels are expressed in the human RPE tissue and that this expression is conserved in the ARPE-19 cell line. Negative controls in which water, instead of template, was used showed no amplification products.

**Figure 3:**
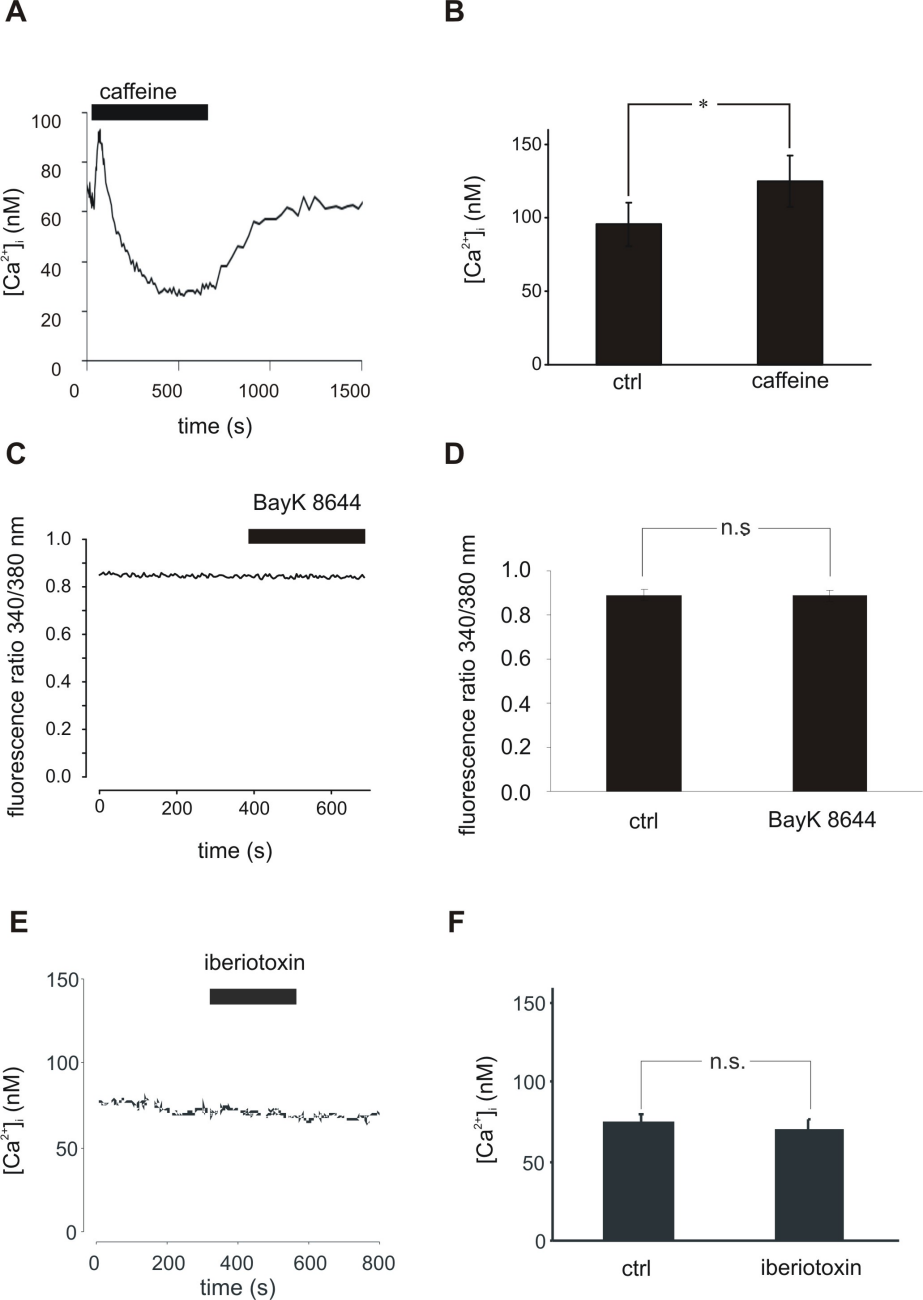
Influence of ryanodine receptor and L-type Ca^2+^ channel activation on [Ca^2+^]_i_ in confluent cultures of ARPE-19 cells. **A:** Application of 50 mM caffeine leads to an instantaneous [Ca^2+^]_i_ increase followed by a sustained decrease. **B:** 50 mM caffeine induced a significant increase in [Ca^2+^]_i_ (p<0.05, n=6). **C:** Activation of L-type Ca^2+^ channels by 5 μM BayK8644 did not influence [Ca^2+^]_i_ levels in ARPE-19 cells. **D:** Mean [Ca^2+^]_i_ did not differ before and during application of 5 μM BayK8644 (n=6). **E:** Inhibition of BK channels by 100 nM iberiotoxin did not influence [Ca^2+^]_i_ levels in ARPE-19 cells. **F:** Mean [Ca^2+^]_i_ did not differ before and during application of 100 nM iberiotoxin (n=5).

**Figure 4:**
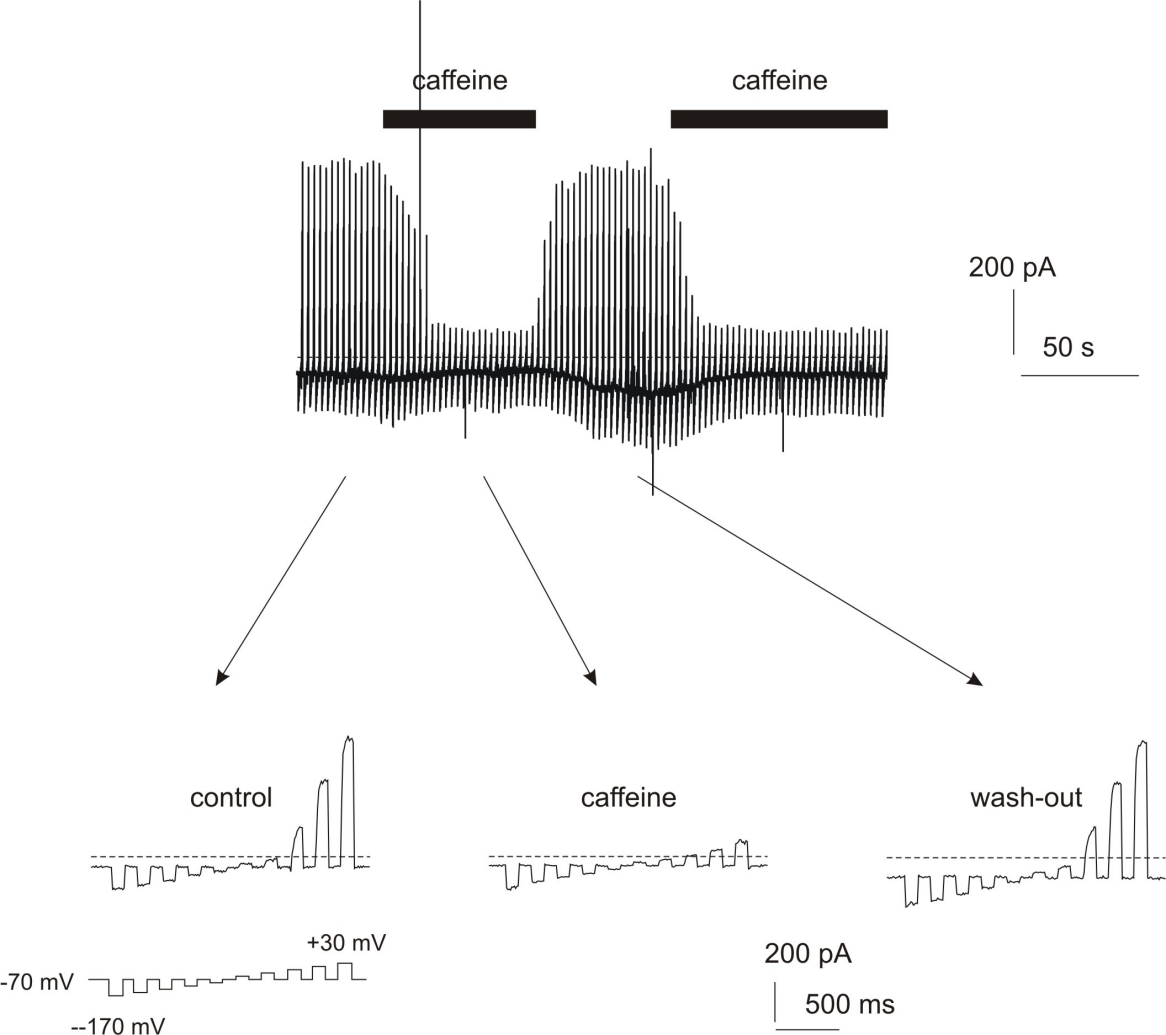
Caffeine-induced changes in whole-cell currents of ARPE-19 cells. To monitor changes in whole-cell currents the following pulse protocol has been used: every 2.5 s the cells were hyperpolarized from a holding potential of −70 mV to five potentials with 20 mV increment for 100 ms. These hyperpolarizations were followed by five depolarizing voltage steps with 20 mV increment for 100 ms. Caffeine led to a strong down-regulation of outward currents in ARPE-19 cells. At the bottom typical current responses before (‘control’), during (‘caffeine’) and after (‘wash-out’) application of 50 mM caffeine are shown.

In other cell types, it has been demonstrated that BK channels are coupled to ryanodine receptors and that the Ca^2+^ released from intracellular Ca^2+^ stores through activation of ryanodine receptors leads to increased activity of BK channels. As the existence of ryanodine receptors in RPE cells has been postulated by binding studies using melanosomes isolated from RPE cells and ^3^H-labeled ryanodine [6], we first tested if activation of ryanodine receptors leads to an increase in [Ca^2+^]_i_ in RPE cells. We used 50 mM caffeine to stimulate ryanodine receptors. In Figure 3A a typical Ca^2+^-response of a confluent ARPE-19 culture to caffeine stimulation is shown. Caffeine led to a significant increase of [Ca^2+^]_i_ of 34.16±10.16% (n=6; Figure 3A,B). This was followed by a sustained decrease in intracellular free Ca^+^ to 41.26±3.4% of the basal Ca^2+^ level before caffeine application (n=5). After washout of caffeine the cytosolic free Ca^2+^ recovered to 83.3±6.2% of the basal Ca^2+^ level measured before application of caffeine (n=5; p>0.001). The initial increase in [Ca^2+^]_i_ induced by application of caffeine should lead to an activation of BK channel, whereas the caffeine-dependent decrease in [Ca^2+^]_i_ should lead to a deactivation of the BK channel. Thus, we investigated changes of membrane currents in ARPE-19 cells by patch-clamp measurements. Surprisingly, BK channels were not activated in response to the application of caffeine; instead, caffeine led to a clear downregulation of outward currents in RPE cells during the phase of reduced Ca^2+^ levels (Figure 4).

**Figure 5:**
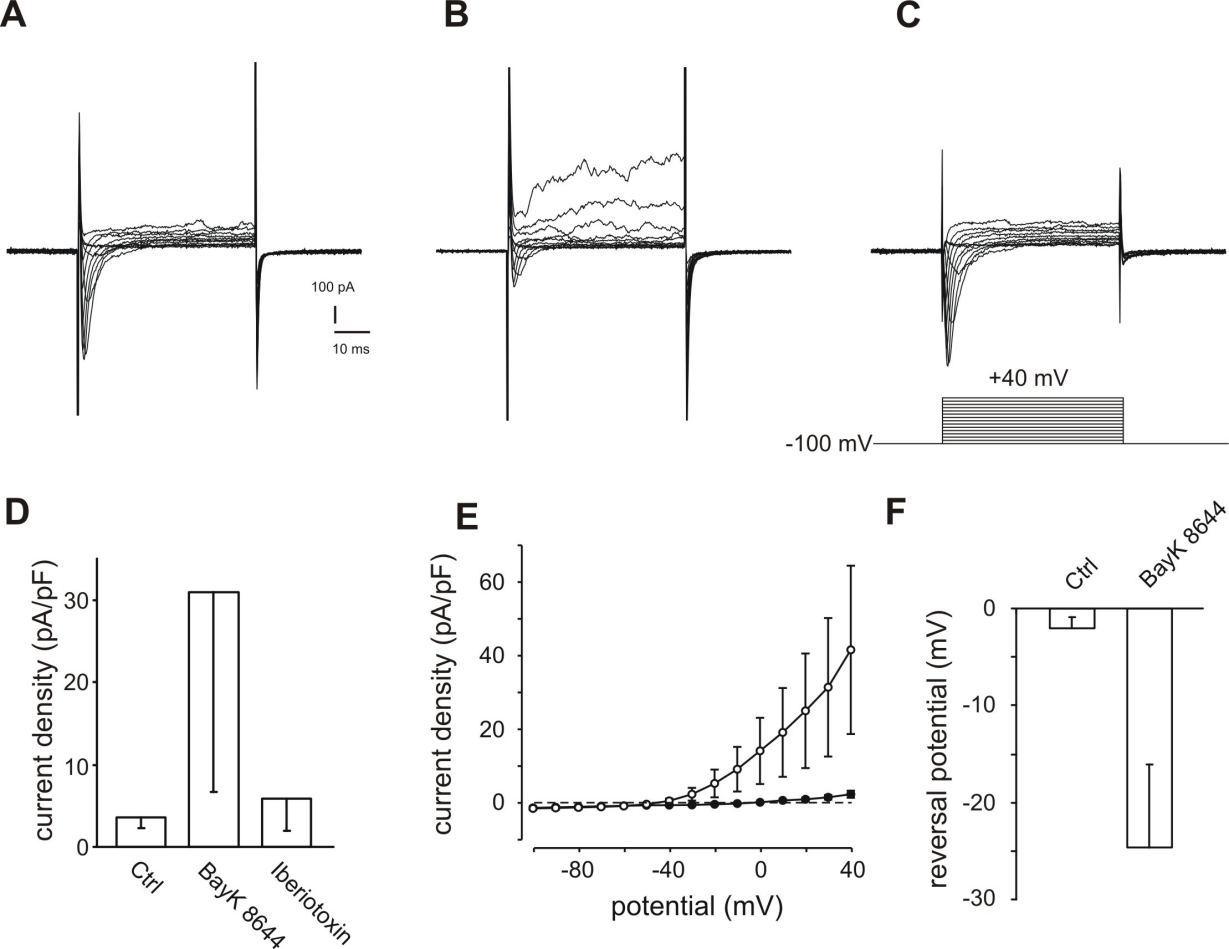
Activation of BK channels in ARPE-19 cells by the BayK 8644-induced activation of L-type Ca^2+^ channels. **A:** Control currents were evoked by 15 depolarizing voltage steps of 50 ms duration with 10 mV increment before application of BayK 8644 (holding potential −100 mV). **B:** The same cell after application of 5 μM BayK 8644 displayed a considerable increase of outward currents. **C:** The BayK 8644-induced outward currents are blocked by the additional application of 100 nM iberiotoxin. Note that in this cell a transient inward current activated by depolarisation of the cell can be seen. This becomes smaller in the presence of BayK8644 (**B**) because the current is counterbalanced by the outward current which became larger. However, when the outward current was blocked by additional application of iberiotoxin (**C**) then the inward current shows in the presence of BayK 8644 an amplitude which is larger (130% of control) than that before application of BayK 8644 (**A**). Thus, this inward current is most likely a current through L-type Ca^2+^ channels. Since comparable strong L-type currents were only rarely observed, this effect of the BayK8644/iberiotoxin application was not further studied. **D:** Mean current densities measured at +40 mV are considerably increased by the application of BayK 8644. Abbreviations: Control current (Ctrl); current densities after application of 5 μM BayK 8644 (BayK 8644); current densities after additional application of 100 nM iberiotoxin (iberiotoxin; n=3). **E:** The comparison of current-voltage relationship of current densities before (filled circles) and after (open circles) application of 5 μM BayK 8644 illustrates the voltage-dependent current increases induced by BAYK 8644 application (n=5). For illustration the zero current is indicated by a dashed line. **F:** BayK 8644 application shifted the reversal potential to negative potentials. The reversal potential was defined as the point of intersection of the I/U-lines with the x-axes. I/U-lines were generated by plotting the maximal currents evoked by the depolarizing steps in the pulse protocol described in **A** against the potentials (n=5).

Another possible source for Ca^2+^ in RPE cells are L-type Ca^2+^ channels, which are known to be functionally expressed in RPE cells. These channels could increase intracellular free Ca^2+^ by direct opening using the L-type channel opener BayK 8644 or by depolarization of the cell membrane potential by blockade of BK channels by iberiotoxin. Neither application of 10 µM BayK 8644 nor application of 100 nM iberiotoxin resulted in changes in intracellular free Ca^2+^ (Figure 3C-F). Although we were not able to detect an BayK 8644-induced increase in [Ca^2+^]_i_ (Figure 3C,D), we found that in 6 of 7 cells treated with BayK 8644, the maximal outward current at +40 mV was significantly increased by at least 35.38%. The current-voltage relationship before and after addition of 5 µM BayK 8644 (Figure 5E) demonstrated the dramatic increase of the outward currents. In addition, the reversal potential was shifted as indicated by the changed zero-current potential (Figure 5E). In one cell the current was increased nearly 16-fold. In addition to the augmentation of outward currents, BayK 8644 shifted the reversal potential from −2±1.15 mV to −24.6±8.52 mV (n=5; Figure 5E,F). Figure 5A-C shows the response of a representative cell. In 3 cells we applied 100 nM iberiotoxin in addition to BayK 8644. The outward current activated by the application of BayK 8644 to 683.73% could be completely blocked by the addition of 100 nM iberiotoxin, a specific BK channel blocker, to 156.23% compared to the initial maximal outward current at +40 mV (Figure 5A,D).

## Discussion

We show here for the first time, that human RPE cells isolated directly from native tissue express transcript for Ca^2+^-dependent BK K^+^ channels. These channels are activated either by depolarization of the cell membrane or by changes in [Ca^2+^]_i_. We demonstrated that they are activated in RPE cells by increasing [Ca^2+^]_i_ and that this Ca^2+^ is provided by voltage-dependent L-type Ca^2+^ channels and not by intracellular Ca^2+^ store depletion through ryanodine receptors.

We investigated the functional properties of BK channels using the ARPE-19 cell line, which shares many components of Ca^2+^ signaling with native RPE cells such as the subtype of L-type channel, TRPC channels, or ryanodine receptors [5,11,13,25]. Thus, although ARPE-19 cells do not fully represent native RPE cells, basic mechanisms which result from the interaction of these Ca^2+^ transporting proteins can be studied and account most likely for the native cells. This is further supported by the observation that not only native RPE cells express BK channels but also this expression is maintained in cell culture by ARPE-19 cells and by rabbit RPE cells in primary culture [23]. A unique property of BK channels is that they may be activated either by depolarization of the cell membrane or by an increase in [Ca^2+^]_i_ [26–28]. In several cell types BK channels have been shown to be directly activated by Ca^2+^ either released from intracellular stores by ryanodine receptors or by entering the cell through voltage-gated Ca^2+^ channels [29–33]. The presence of ryanodine receptors in RPE cells has been shown previously by binding studies with radiolabeled ryanodine and melanosomes isolated from the RPE [6] and by the reduction of a mechanically induced increase in [Ca^2+^]_i_ by ryanodine [34]. We could confirm the presence of functional ryanodine receptors as they were activated by the addition of caffeine (Figure 3A,B). Additionally, several studies have demonstrated the presence of voltage-gated Ca^2+^ channels in the RPE [7–11,18]. The stimulation of both ryanodine receptors or voltage-gated Ca^2+^ channels leads to an increase in [Ca^2+^]_i_ and thereby potentially to an activation of Ca^2+^-activated BK channels. We show here that the caffeine-induced Ca^2+^ released from intracellular stores is not sufficient for the opening of BK channels. To identify other activators of BK channel activity, we applied caffeine to empty ryanodine-sensitive Ca^2+^ stores. This application led to an initial rise of intracellular free Ca^2+^, which was followed by a sustained decrease to a level below the resting Ca^2+^ level. The initial rise is likely due to depletion of ryanodine-sensitive Ca^2+^ stores, whereas the following decrease in Ca^2+^ results from activation of plasma membrane Ca^2+^-ATPase and Na^+^/Ca^2+^ exchanger [35,36]. A volume-dependent change in the BK channel activity is unlikely because caffeine did not change the cell volume or an increase in extracellular osmolarity did not change intracellular free Ca^2+^ (see Methods). However, the analysis of BK channel activity during caffeine application revealed that the initial rise did not increase BK channel activity whereas in the phase of reduced Ca^2+^ levels the activity of BK channels was subsequently reduced. Since the resting Ca^2+^ level results from the balance of Ca^2+^-ATPase activity and TRPC channels activity, it is likely that the basal activity of BK channels depends on the activity of TRPC channels [13]. Alternatively, activation of L-type Ca^2+^ channels by the specific agonist BayK 8644 did not lead to an general increase in [Ca^2+^]_i_. Consequently, with our setup for measurements of [Ca^2+^]_i_, we could not detect changes in [Ca^2+^]_i_, though we have shown previously that application of BayK 8644 leads to a strong activation of L-type Ca^2+^ currents [17,18,37]. Nevertheless, opening of L-type Ca^2+^ channels led to a sizeable activation of BK channels in RPE cells. This can be seen by a shift of the zero-current potential of whole-current from –2 to –24.6 mV. Under our recording conditions, the resting zero-current potential was rather positive compared to that detected in other studies [38,39]. This is because, in contrast to these studies, we used equal intracellular and extracellular Cl^-^ concentrations. Due to the rather large Cl^-^ conductance and the small inward rectifier conductance of ARPE-19 cells, this results in a zero-current potential close to zero. However, the shift of the zero-current potential toward more negative membrane voltages clearly indicates the activation of a K^+^ conductance. From studies on neurons it is known that opening of Ca^2+^ channels do not lead to a uniform increase in [Ca^2+^]_i_ in the whole cell. Instead, the Ca^2+^ signal from one Ca^2+^ channel creates a nanodomain, and as a result, clusters of Ca^2+^ channels form microdomains [40]. As a consequence, the Ca^2+^ signals strongly depend on the distance from the Ca^2+^ channel. At a distance of 200 nm they are already 10-fold smaller than at a 20 nm distance from the Ca^2+^ channel [41]. A change in voltage-dependence of BK channels by approximately −25 mV, as we observed in RPE cells, needs a strong increase in [Ca^2+^]_i_ that is only obtained in close vicinity to Ca^2+^ channels. Recently, a direct interaction between the α subunits of voltage-gated Ca^2+^ channels and BK channels has been demonstrated [42,43]. Activation of ryanodine receptors failed to activate BK channels, while Ca^2+^ entry through Ca^2+^ channels did activate BK channels in RPE cells. Therefore, it seems likely that in RPE cells, both latter channels interact physically, as has been shown for brain tissues and heterologously expressed channels [42,43]. However, the colocalization of L-type channels and BK channels in the basolateral membrane of native RPE cells needs to be demonstrated to verify this conclusion.

### Physiologic implications

The question arises whether functional L-type/BK channel interaction plays a role for RPE function. As mentioned in the previous section, native RPE cells express both L-type channels and BK channels. Thus, their functional interaction in native cells is likely. The next question would be how these ion channels may contribute to cellular functions, as they show activation thresholds which are far more positive than the resting potential of RPE cells of −50 mV. The L-type channels, which are expressed in RPE cells, are the channels of the Ca_V_1.3 subtype, which can show activation thresholds as negative as −40 mV [44]. Furthermore, in an earlier study we demonstrated that L-type channels can contribute to changes in intracellular free Ca^2+^ at fixed membrane potentials which are more negative than the activation threshold of these channels. This is enabled by a phosphorylation-dependent shift of the voltage-dependent activation of these toward more negative values. This results in an increase in the number of active channels at this potential, leading to an increase in intracellular free Ca^2+^ [45]. That BK channels can be active in RPE cells at physiologic membrane voltages has been shown by two studies exploring the activity of BK channels in response to increases in intracellular free Ca^2+^ [22,23]. When BK channels are activated by this maneuver, they show a voltage-dependence in the range of the resting potential of RPE cells. This effect is due to the Ca^2+^-dependent voltage-dependence of BK channels. Increases in intracellular free Ca^2+^ shifts the voltage-dependence toward more negative values [28]. In contrast to the studies by Sheu et al. [22] and Tao et al. [23], we showed an increase in BK channels without a general in increase in the cytosolic Ca^2+^. Thus, in this study, the activation threshold of BK channels remained very positive. However, in native cells both increase of L-type channel activity and increase in cytosolic free Ca^2+^ would lead to considerable increase in BK channel activity at physiologic membrane voltages. Even in this study, BK channel activation led to a shift of the zero-current potential by −25 mV. The functions for BK channels in RPE cells that have been described so far mainly concern water and ion transport through the RPE [21–23]. They have been shown to be influenced by different stimuli. It has been reported that BK channels in RPE cells are inhibited by oxidizing agents [20], and activated by exposure to hypotonic solutions [21] and by stimulating the membrane mechanically [22]. The latter stimulation by stretching the membrane seems to be independent of [Ca^2+^]_i_ since it is maintained in inside-out patches exposed to different Ca^2+^ concentrations [22].

In this study, we provide data for an alternative mechanism for BK channel activation by possible direct coupling to voltage-gated Ca^2+^ channels. Voltage-gated Ca^2+^ channels have been shown to be involved in different functions of the RPE. Long exposure of dark-adapted eyes to a light stimulus leads to slowly rising signal in the electrooculogram, the so-called light peak. This light peak is reduced by application of the L-type Ca^2+^ channel antagonist nimodipine, indicating that L-type Ca^2+^ channels are activated by light exposure [46,47]. The coactivation of L-type channels and BK channels suggests a possible role for BK channels in this mechanism.

In neurons, BK channels provide a negative feedback mechanism for Ca^2+^ channel-induced neurotransmitter release [1]. As L-type Ca^2+^ channels in RPE are responsible for Ca^2+^-induced secretion of vascular endothelial growth factor [19], it seems likely that BK channels analogously control this secretion in the RPE.

## References

[1] Wang ZW, Saifee O, Nonet ML, Salkoff L (2001). SLO-1 potassium channels control quantal content of neurotransmitter release at the C. elegans neuromuscular junction.. Neuron.

[2] Jin W, Sugaya A, Tsuda T, Ohguchi H, Sugaya E (2000). Relationship between large conductance calcium-activated potassium channel and bursting activity.. Brain Res.

[3] Brenner R, Peréz GJ, Bonev AD, Eckman DM, Kosek JC, Wiler SW, Patterson AJ, Nelson MT, Aldrich RW (2000). Vasoregulation by the beta1 subunit of the calcium-activated potassium channel.. Nature.

[4] Solaro CR, Prakriya M, Ding JP, Lingle CJ (1995). Inactivating and noninactivating Ca(2+)- and voltage-dependent K+ current in rat adrenal chromaffin cells.. J Neurosci.

[5] Wimmers S, Karl MO, Strauss O (2007). Ion channels in the RPE.. Prog Retin Eye Res.

[6] Salceda R, Sanchez-Chavez G (2000). Calcium uptake, release and ryanodine binding in melanosomes from retinal pigment epithelium.. Cell Calcium.

[7] Ueda Y, Steinberg RH (1993). Voltage-operated calcium channels in fresh and cultured rat retinal pigment epithelial cells.. Invest Ophthalmol Vis Sci.

[8] Ueda Y, Steinberg RH (1995). Dihydropyridine-sensitive calcium currents in freshly isolated human and monkey retinal pigment epithelial cells.. Invest Ophthalmol Vis Sci.

[9] Strauss O, Wienrich M (1994). Ca(2+)-conductances in cultured rat retinal pigment epithelial cells.. J Cell Physiol.

[10] Rosenthal R, Strauss O (2002). Ca2+-channels in the RPE.. Adv Exp Med Biol.

[11] Wimmers S, Coeppicus L, Rosenthal R, Strauss O (2008). Expression profile of voltage-dependent Ca2+ channel subunits in the human retinal pigment epithelium.. Graefes Arch Clin Exp Ophthalmol.

[12] Bollimuntha S, Cornatzer E, Singh BB (2005). Plasma membrane localization and function of TRPC1 is dependent on its interaction with beta-tubulin in retinal epithelium cells.. Vis Neurosci.

[13] Wimmers S, Strauss O (2007). Basal calcium entry in retinal pigment epithelial cells is mediated by TRPC channels.. Invest Ophthalmol Vis Sci.

[14] Ryan JS, Baldridge WH, Kelly ME (1999). Purinergic regulation of cation conductances and intracellular Ca2+ in cultured rat retinal pigment epithelial cells.. J Physiol.

[15] Kuriyama S, Ohuchi T, Yoshimura N, Honda Y (1991). Growth factor-induced cytosolic calcium ion transients in cultured human retinal pigment epithelial cells.. Invest Ophthalmol Vis Sci.

[16] Heth CA, Marescalchi PA (1994). Inositol triphosphate generation in cultured rat retinal pigment epithelium.. Invest Ophthalmol Vis Sci.

[17] Strauss O, Buss F, Rosenthal R, Fischer D, Mergler S, Stumpff F, Thieme H (2000). Activation of neuroendocrine L-type channels (alpha1D subunits) in retinal pigment epithelial cells and brain neurons by pp60(c-src).. Biochem Biophys Res Commun.

[18] Rosenthal R, Thieme H, Strauss O (2001). Fibroblast growth factor receptor 2 (FGFR2) in brain neurons and retinal pigment epithelial cells act via stimulation of neuroendocrine L-type channels (Ca(v)1.3).. FASEB J.

[19] Rosenthal R, Heimann H, Agostini H, Martin G, Hansen LL, Strauss O (2007). Ca2+ channels in retinal pigment epithelial cells regulate vascular endothelial growth factor secretion rates in health and disease.. Mol Vis.

[20] Sheu SJ, Wu SN (2003). Mechanism of inhibitory actions of oxidizing agents on calcium-activated potassium current in cultured pigment epithelial cells of the human retina.. Invest Ophthalmol Vis Sci.

[21] Sheu SJ, Wu SN, Hu DN, Chen JF (2004). The influence of hypotonicity on large-conductance calcium-activated potassium channels in human retinal pigment epithelial cells.. J Ocul Pharmacol Ther.

[22] Sheu SJ, Wu SN, Hu DN (2005). Stretch-stimulated activity of large conductance calcium-activated potassium channels in human retinal pigment epithelial cells.. J Ocul Pharmacol Ther.

[23] Tao Q, Kelly ME (1996). Calcium-activated potassium current in cultured rabbit retinal pigment epithelial cells.. Curr Eye Res.

[24] Grynkiewicz G, Poenie M, Tsien RY (1985). A new generation of Ca2+ indicators with greatly improved fluorescence properties.. J Biol Chem.

[25] Reigada D, Lu W, Mitchell CH (2006). Glutamate acts at NMDA receptors on fresh bovine and on cultured human retinal pigment epithelial cells to trigger release of ATP.. J Physiol.

[26] Wolfe JT, Wang H, Howard J, Garrison JC, Barrett PQ (2003). T-type calcium channel regulation by specific G-protein betagamma subunits.. Nature.

[27] Salkoff L, Butler A, Ferreira G, Santi C, Wei A (2006). High-conductance potassium channels of the SLO family.. Nat Rev Neurosci.

[28] Latorre R, Brauchi S (2006). Large conductance Ca2+-activated K+ (BK) channel: activation by Ca2+ and voltage.. Biol Res.

[29] Kang TM, So I, Kim KW (1995). Caffeine- and histamine-induced oscillations of K(Ca) current in single smooth muscle cells of rabbit cerebral artery.. Pflugers Arch.

[30] Martinez-Pinna J, McLachlan EM, Gallego R (2000). Distinct mechanisms for activation of Cl- and K+ currents by Ca2+ from different sources in mouse sympathetic neurones.. J Physiol.

[31] Paltauf-Doburzynska J, Frieden M, Spitaler M, Graier WF (2000). Histamine-induced Ca2+ oscillations in a human endothelial cell line depend on transmembrane ion flux, ryanodine receptors and endoplasmic reticulum Ca2+-ATPase.. J Physiol.

[32] Evans MG, Lagostena L, Darbon P, Mammano F (2000). Cholinergic control of membrane conductance and intracellular free Ca2+ in outer hair cells of the guinea pig cochlea.. Cell Calcium.

[33] Zemkova H, Vanecek J (2000). Differences in gonadotropin-releasing hormone-induced calcium signaling between melatonin-sensitive and melatonin-insensitive neonatal rat gonadotrophs.. Endocrinology.

[34] Stalmans P, Himpens B (1997). Confocal imaging of Ca2+ signaling in cultured rat retinal pigment epithelial cells during mechanical and pharmacologic stimulation.. Invest Ophthalmol Vis Sci.

[35] Kennedy BG, Mangini NJ (1996). Plasma membrane calcium-ATPase in cultured human retinal pigment epithelium.. Exp Eye Res.

[36] Mangini NJ, Haugh-Scheidt L, Valle JE, Cragoe EJ, Ripps H, Kennedy BG (1997). Sodium-calcium exchanger in cultured human retinal pigment epithelium.. Exp Eye Res.

[37] Mergler S, Steinhausen K, Wiederholt M, Strauss O (1998). Altered regulation of L-type channels by protein kinase C and protein tyrosine kinases as a pathophysiologic effect in retinal degeneration.. FASEB J.

[38] Wen R, Lui GM, Steinberg RH (1993). Whole-cell K+ currents in fresh and cultured cells of the human and monkey retinal pigment epithelium.. J Physiol.

[39] Hughes BA, Takahira M (1996). Inwardly rectifying K+ currents in isolated human retinal pigment epithelial cells.. Invest Ophthalmol Vis Sci.

[40] Augustine GJ, Santamaria F, Tanaka K (2003). Local calcium signaling in neurons.. Neuron.

[41] Neher E (1998). Vesicle pools and Ca2+ microdomains: new tools for understanding their roles in neurotransmitter release.. Neuron.

[42] Hansen RS, Diness TG, Christ T, Demnitz J, Ravens U, Olesen SP, Grunnet M (2006). Activation of human ether-a-go-go-related gene potassium channels by the diphenylurea 1,3-bis-(2-hydroxy-5-trifluoromethyl-phenyl)-urea (NS1643).. Mol Pharmacol.

[43] Berkefeld H, Sailer CA, Bildl W, Rohde V, Thumfart JO, Eble S, Klugbauer N, Reisinger E, Bischofberger J, Oliver D, Knaus HG, Schulte U, Fakler B (2006). BKCa-Cav channel complexes mediate rapid and localized Ca2+-activated K+ signaling.. Science.

[44] Koschak A, Reimer D, Huber I, Grabner M, Glossmann H, Engel J, Striessnig J (2001). alpha 1D (Cav1.3) subunits can form l-type Ca2+ channels activating at negative voltages.. J Biol Chem.

[45] Mergler S, Strauss O (2002). Stimulation of L-type Ca(2+) channels by increase of intracellular InsP3 in rat retinal pigment epithelial cells.. Exp Eye Res.

[46] Wollmann G, Lenzner S, Berger W, Rosenthal R, Karl MO, Strauss O (2006). Voltage-dependent ion channels in the mouse RPE: comparison with Norrie disease mice.. Vision Res.

[47] Marmorstein LY, Wu J, McLaughlin P, Yocom J, Karl MO, Neussert R, Wimmers S, Stanton JB, Gregg RG, Strauss O, Peachey NS, Marmorstein AD (2006). The light peak of the electroretinogram is dependent on voltage-gated calcium channels and antagonized by bestrophin (best-1).. J Gen Physiol.

